# A Combined Metabolomic and Metagenomic Approach to Discriminate Raw Milk for the Production of Hard Cheese

**DOI:** 10.3390/foods10010109

**Published:** 2021-01-06

**Authors:** Paolo Bellassi, Gabriele Rocchetti, Marco Nocetti, Luigi Lucini, Francesco Masoero, Lorenzo Morelli

**Affiliations:** 1 Department for Sustainable Food Process—DiSTAS, Università Cattolica del Sacro Cuore, Via Emilia Parmense 84, 29122 Piacenza, Italy; paolo.bellassi@unicatt.it (P.B.); luigi.lucini@unicatt.it (L.L.); lorenzo.morelli@unicatt.it (L.M.); 2 Primary Production Department, Parmigiano Reggiano Cheese Consortium, Via J. F. Kennedy 18, 42124 Reggio Emilia, Italy; nocetti@parmigianoreggiano.it; 3 Department of Animal Science, Food and Nutrition—DiANA, Università Cattolica del Sacro Cuore, Via Emilia Parmense 84, 29122 Piacenza, Italy; francesco.masoero@unicatt.it

**Keywords:** milk quality, feeding systems, foodomics, lipids, secondary metabolites

## Abstract

The chemical composition of milk can be significantly affected by different factors across the dairy supply chain, including primary production practices. Among the latter, the feeding system could drive the nutritional value and technological properties of milk and dairy products. Therefore, in this work, a combined foodomics approach based on both untargeted metabolomics and metagenomics was used to shed light onto the impact of feeding systems (i.e., hay vs. a mixed ration based on hay and fresh forage) on the chemical profile of raw milk for the production of hard cheese. In particular, ultra-high-performance liquid chromatography quadrupole time-of-flight mass spectrometry (UHPLC-QTOF) was used to investigate the chemical profile of raw milk (n = 46) collected from dairy herds located in the Po River Valley (Italy) and considering different feeding systems. Overall, a total of 3320 molecular features were putatively annotated across samples, corresponding to 734 unique compound structures, with significant differences (*p* < 0.05) between the two feeding regimens under investigation. Additionally, supervised multivariate statistics following metabolomics-based analysis allowed us to clearly discriminate raw milk samples according to the feeding systems, also extrapolating the most discriminant metabolites. Interestingly, 10 compounds were able to strongly explain the differences as imposed by the addition of forage in the cows’ diet, being mainly glycerophospholipids (i.e., lysophosphatidylethanolamines, lysophosphatidylcholines, and phosphatidylcholines), followed by 5-(3′,4′-Dihydroxyphenyl)-gamma-valerolactone-4′-*O*-glucuronide, 5a-androstan-3a,17b-diol disulfuric acid, and N-stearoyl glycine. The markers identified included both feed-derived (such as phenolic metabolites) and animal-derived compounds (such as lipids and derivatives). Finally, although characterized by a lower prediction ability, the metagenomic profile was found to be significantly correlated to some milk metabolites, with *Staphylococcaceae*, *Pseudomonadaceae*, and *Dermabacteraceae* establishing a higher number of significant correlations with the discriminant metabolites. Therefore, taken together, our preliminary results provide a comprehensive foodomic picture of raw milk samples from different feeding regimens, thus supporting further ad hoc studies investigating the metabolomic and metagenomic changes of milk in all processing conditions.

## 1. Introduction

Metabolomics is the comprehensive study of the low-molecular-weight compounds (<1500 Da) involved in metabolism, including carbohydrates, lipids, amino acids, biogenic amines, and organic acids [1]. Overall, due to the important role of metabolism across all biological processes, metabolomics-based studies have been increasingly used to understand the physiological processes associated with economically important traits in the dairy industry, such as milk production in dairy cattle (as affected by the feeding systems) and cheese manufacturing [2,3,4]. Additionally, several metabolomic research efforts have been mainly devoted to a better understanding of the impact of diet on rumen health in dairy cows [5,6].

There are several factors across the dairy supply chain including primary production practices (such as feeding systems, housing, breeding, animal health and welfare) and processing conditions that can significantly affect the composition, quality, and functionality of milk, dairy products, and ingredients [7,8,9]. Accordingly, enhanced technologies in the field of “foodomics” have also increased our understanding of factors affecting the composition of milk beyond that of traditional analytical chemistry methods, with implications for the prediction of products’ functionality, digestibility, and authentication [10,11]. These approaches (mainly targeted) have been exploited by several researchers to evaluate the impact of different ration types on several parameters, such as milk production and its composition (focusing mainly on changes in lipidomic profiles) [12].

Additionally, regarding the transformation of milk in hard cheese, it is important to consider the starting microbiota composition. In this regard, members of the milk microbiota (including lactic acid bacteria) could directly affect the quality of milk intended for processing into cheese [13]. In particular, some specific dominant bacteria play an important role in udder homeostasis, which guarantees high milk quality [14]. Furthermore, the raw milk microbiota could be deeply influenced by specific aspects of the farm environment, including the teat surface, air, dust, barn conditions, and milking parlor environment, which are responsible for milk contamination [15,16]. Overall, it is fairly accepted that the microbial community in milk farm samples differs significantly from that of dairy plant samples; in this regard, according to the literature, psychrotolerant communities (mainly *Pseudomonadales*) present in farm samples can proliferate during milk storage at refrigeration temperature [17,18]. Furthermore, *Pseudomonas* spp. counts can be related to the cleaning and sanitation procedures of milking equipment and to water quality [18].

To the best of our knowledge, scarce information is available in the scientific literature about the potential correlations between the metabolomic and metagenomic profiles of raw milk used for hard cheese manufacturing, as affected by feeding regimens. Therefore, in this work, an ultra-high-performance liquid chromatography coupled with quadrupole-time of flight mass spectrometry (UHPLC-QTOF-MS), followed by univariate and multivariate statistical approaches, was used to compare the metabolomic and metagenomic profiles of milk samples, collected from dairy farms in which cows were fed different ration types—hay (H-FS) and a mixed ration (including hay and grass fresh forage; MR-FS). Additionally, we aimed at identifying discriminant milk metabolites that were specific of each feeding regimen condition under investigation, and likely correlated to the microbial profile of raw milk used for the cheese-making process. In this regard, the high-resolution and comprehensive approach could be of particular interest for the dairy industry to evaluate the quality and traceability of dairy products.

## 2. Materials and Methods

### 2.1. Sample Collection

In this work, we analyzed a total of 46 milk samples collected from dairy herds located in the Protected Designation of Origin (PDO) area of Parmigiano Reggiano (Po River Valley, Italy) for detailed metabolomic analyses. In this regard, we sampled bulk milk from storage tanks at 18 °C. Thereafter, 500 mL of milk samples were collected in sterile glass bottles, frozen, and stored on ice during transportation to the laboratory at Università Cattolica del Sacro Cuore (Piacenza, Italy). The milk samples were classified according to the feeding regimen declared by each herd, being hay-based (H-FS; 29 samples) and fresh forage/hay-based (MR-FS; 17 samples).

### 2.2. Extraction of Milk Metabolites

The extraction of milk metabolites was carried out as previously reported by Xi et al. [19], with minor modifications [7]. Briefly, milk samples were skimmed according to a centrifugation at 4500× *g* for 10 min at 4 °C, and the skim milk was frozen at −80 °C for further processing. Thereafter, the 46 skim milk samples were thawed and thoroughly vortex mixed. An aliquot of 2 mL of each sample was added to 14 mL of acetonitrile (LC-MS grade, Sigma-Aldrich, Madison, CA, USA) acidified with 3% formic acid, mixed by vortexing for 2 min and processed with ultrasounds for 5 min. Next, the samples were centrifuged at 12,000× *g* for 15 min at 4 °C to remove large biomolecules (such as proteins). The supernatants were then filtered through a 0.20-μm cellulose syringe filter in amber vials until the further metabolomic analysis.

### 2.3. Untargeted Metabolomics Based on UHPLC-QTOF Mass Spectrometry

In this work, the untargeted metabolomic analysis was based on an ultra-high-pressure liquid chromatographic system (Agilent 1200 series) coupled with a quadrupole time-of-flight mass spectrometer (Agilent 6550 iFunnel), as previously reported [7,10]. Overall, each prepared milk extract (i.e., belonging to both H-FS and MR-FS groups) was analyzed in positive polarity (ESI+) using a Full Scan in the m/z range 100–1200 (0.8 spectra/s), in extended dynamic range mode with a nominal mass resolution of 30,000 FWHM. The chromatography was based on a water-acetonitrile (both LC-MS grade, from Sigma-Aldrich, Milan, Italy) gradient elution (6–94% acetonitrile in 35 min) with 0.1% formic acid as a phase modifier, using an Agilent Zorbax Eclipse Plus C18 column (50 × 2.1 mm, 1.8 μm). The electrospray conditions were previously optimized [7]. The injection volume was 5 μL, in triplicate for each sample; the injection sequence was randomized, and pooled quality control samples (QCs) were injected at the beginning of the sequence and every 10 sample injections. QCs were acquired using a data-dependent auto-MS/MS mode using 12 precursors per cycle (1 Hz, 50–1200 *m*/*z*, positive polarity, active exclusion after 2 spectra), with collision energies of 10, 20, and 40 eV for collision-induced decomposition.

Thereafter, the collected UHPLC-HRMS data (.d files from Agilent) were converted into .abf files using the Reifycs Abf Converter and then further processed using the software MS-DIAL (version 4.28) [20]. In this regard, automatic peak finding, LOWESS normalization, and annotation via spectral matching (against the database Mass Bank of North America) were performed. The mass range 100–1200 *m*/*z* was searched for peaks with a minimum peak height of 1000 cps for ESI+ polarity. The MS and MS/MS tolerance for peak centroiding was set to 0.01 and 0.05 Da, respectively. Retention time information was excluded from calculation of the total score. Accurate mass tolerance for identification was 0.01 Da for MS and 0.05 Da for MS/MS. The identification step was based on mass accuracy, isotopic pattern, and spectral matching. Gap filling using the peak finder algorithm was performed to fill in missing peaks, considering 5 ppm tolerance for m/z values. Furthermore, the software MS-Finder was used to run in silico prediction of unknown structures, according to the in-house databases [21]. Additionally, in order to achieve a more comprehensive picture of the changes in milk metabolites between the 2 different feeding systems, the raw spectral data were processed according to the targeted “find-by-formula” algorithm, using Agilent Profinder B.06 (Agilent Technologies) software. The highest confidence in annotation was obtained combining monoisotopic mass information with isotopes profile (both isotopic spacing and ratio) and adopting a 5-ppm tolerance for mass accuracy. To this end, the comprehensive Bovine Metabolome Database (BMDB) [22] was used as reference for annotation. Data pre-processing (mass and retention time alignment) and compounds filtering were performed in Agilent Profinder B.06 software: compounds passing mass accuracy (5-ppm) and frequency of detection (within 100% of replications in at least one treatment) thresholds, and having a plausible chromatogram peak feature, were retained and finally used for multivariate statistical analysis. Overall, Level 2 of identification in the untargeted workflow was achieved, as reported by COSMOS Metabolomics Standards Initiative [23]. Additionally, according to Foroutan et al. [12], we considered the term “metabolite species” mainly referring to those molecules with non-unique chemical formulas or masses (e.g., lipids and derivatives), while “unique compound structures” were those compounds possessing a unique chemical formula or mass.

### 2.4. DNA Extraction and Sequencing

Thawed milk samples were mixed thoroughly and centrifuged at 8000× *g* for 10 min. Supernatant was discarded and genomic DNA was isolated from the remaining pellet using the DNeasy^®^ PowerFood^®^ Microbial Kit. The amount of extracted DNA was quantified by using the Qubit™ dsDNA HS Assay Kit (Invitrogen, Carlsbad, CA, USA) and quality was checked on 1% agarose electrophoresis gel. The V3-V4 hypervariable regions of the 16S rRNA gene was sequenced by the sequencing facility for the 16S rRNA gene amplicon using 341F (5′CCTACGGGNGGCWGCAG 3′) and 805R (5′GACTACHVGGGTATC TAATCC 3′) universal primer [24]. Additionally, Illumina MiSeq technology (2 × 300 bp) was used as sequencing platform.

### 2.5. Sequencing Data Processing and Statistical Analysis

Sequencing data were analyzed using MG-RAST (v3.6) and SEED2 (2.1.1) pipeline [25,26]. We obtained a total of 18.61 million raw sequences. The phase of demultiplexing and merging of reverse and forward was conducted by the facility through proprietary scripts. Subsequently, the reads divided into single files per sample were processed using the SEED 2 software and filtered using a reads quality cut off of 25 and eliminating the shorter sequences of 400 bp; all reads were cut at 400 bp. The cleaning of the chimeric sequences was performed using the UPARSE tool embedded in the SEED 2 software package. Additionally, for taxonomic identification and the construction of an operational taxonomic unit (OTU) table, the online platform MG-RAST for taxonomic and phylogenetic profiling was used. MG-RAST annotations were made against Ribosomal Database Project (RDP) [27] with a minimum value of 1E-5 and a minimum identity of 97%. To minimize the effect of non-uniform sequencing depth, each sample was rarefied to 40,549 reads per sample for subsequent analysis. Statistical analysis was conducted using MicrobiomeAnalyst [28]. The alpha diversity was calculated using several indices including: CHAO1, OBSERVED, SHANNON. Meanwhile, beta diversity was calculated by building a PCoA diversity matrix using the Bray–Curtis Index.

Finally, an LDA Effective Size (LEfSe) analysis was conducted that integrates statistical significance with the estimation of biological consistency (effect size). In particular, it first performs the non-parametric factorial Kruskal–Wallis (KW) sum-rank test to detect the characteristics with significant differential abundance with respect to the class of interest, followed by Linear Discriminant Analysis to estimate the effect size of each differentially abundant characteristic.

The sequences files are available in the Sequence Read Archive under accession number: PRJNA685194.

### 2.6. Multivariate Analysis on Metabolomics-Based Data

The multivariate statistical elaboration of metabolomics-based data was performed using two different programs, namely MetaboAnalyst [29] and SIMCA 13 (Umetrics, Malmo, Sweden). Briefly, after data normalization [7], both unsupervised and supervised multivariate statistics were carried out based on principal component analysis (PCA) and orthogonal projections to latent structures discriminant analysis (OPLS-DA), respectively. Additionally, the OPLS-DA model validation parameters (goodness-of-fit R^2^Y together with goodness-of-prediction Q^2^Y) were inspected, considering a Q^2^Y prediction ability of >0.5 as the acceptability threshold [30]. Thereafter, the OPLS-DA model produced was inspected for outliers and permutation testing (*N* > 100) was performed to exclude model over-fitting. The importance of each milk metabolite for discrimination purposes (i.e., when considering the two different feeding systems) was then calculated according to the variable selection method VIP (i.e., variables importance in projection), considering as the minimum significant threshold those values higher than 1.

As the next step, volcano plots were produced for the comparison of H-FS vs. MR-FS by coupling fold-change analysis (cut-off value > 1.2) and ANOVA (*p* < 0.05; post hoc test: Tukey HSD; multiple testing correction: Bonferroni Family-Wise Error Rate). Furthermore, to validate the discriminant VIP markers proposed, Receiver Operating Characteristics (ROC) curves were extrapolated, using the software MetaboAnalyst [31]. To this aim, the area under the ROC curve (AUC) was inspected to evaluate the global performance of each discriminant marker proposed. Finally, a misclassification table made in the software SIMCA 13 was used to classify the prediction dataset observations, thus displaying the overall class prediction accuracy of the OPLS-DA model.

## 3. Results and Discussion

### 3.1. Multivariate Statistical Discrimination of the Different Milk Samples

In this work, an untargeted metabolomics-based approach was used to comprehensively investigate those metabolites better characterizing and discriminating the collected milk samples according to the feeding regimen. In particular, we used an untargeted screening based on UHPLC-ESI-QTOF mass spectrometry combined with both unsupervised and supervised multivariate statistical analyses. Following data acquisition, it was possible to identify a wide variety of chemical classes according to the recently developed and comprehensive “Bovine Metabolome Database” (BMDB) (Foroutan et al., 2020). The Bovine BMDB is a freely available electronic database containing detailed information about small molecule metabolites found in bovines. It was intended to be used for understanding more about bovine biology and the micronutrients found in bovine tissues and biofluids as well as improving veterinary care for beef and dairy cattle.

Overall, a total of 3320 molecular features were putatively annotated across samples. However, considering the exclusive mass spectra of each compound (i.e., excluding compound isomers), the number was reduced to 561 metabolite species. Furthermore, a dedicated tandem-MS approach using pooled quality control (QC) samples allowed us to record an additional 173 unique structures by using the comprehensive database MoNA (Mass Bank of North America), provided by MS-Dial software. The wide number of chemical features obtained by using this annotation workflow is consistent with the overall complexity of the food matrix (i.e., milk) under investigation. In particular, a large number of isomeric forms of lipids were observed, as reported in previous works [7,12]. The entire list of milk metabolites annotated according to our analytical workflow is provided as Supplementary Material, together with their MS-only and MS/MS spectra. Overall, the compounds annotated mainly belonged to lipids (such as glycerolipids, glycerophospholipids, lysophospholipids, and triglycerides), followed by amino acids and derivatives, and secondary metabolites (likely related to carry-over processes). Additionally, the MS/MS approach allowed us to confirm the structural identity of some metabolites among the others, such as stearic acid, palmitoyl glucuronide, SM(d18:1/12:0), and other phospholipids (Supplementary Material).

**Figure 1:**
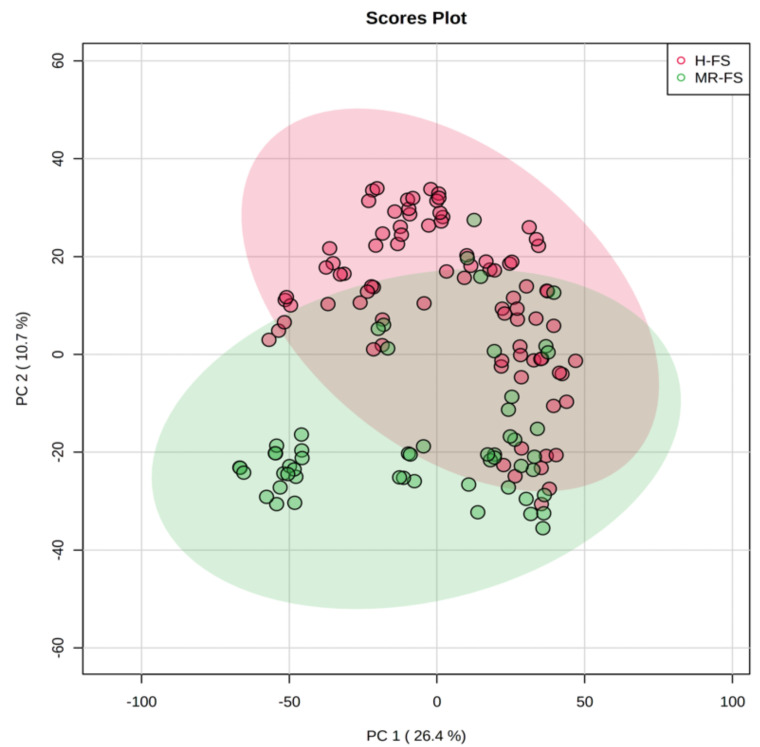
Unsupervised principal component analysis considering the milk samples from different feeding regimens (i.e., H-FS: hay-based feeding system; MR-FS: fresh forage/hay-based feeding system). The different colored ellipses represent milk samples grouped according to the declared feeding regimen.

Thereafter, multivariate statistics based on both unsupervised and supervised statistics was carried out in order to group samples according to their similarity in metabolomic composition. Firstly, principal component analysis (PCA) was carried out, and the corresponding bidimensional score plot is reported as Figure 1.

**Figure 2:**
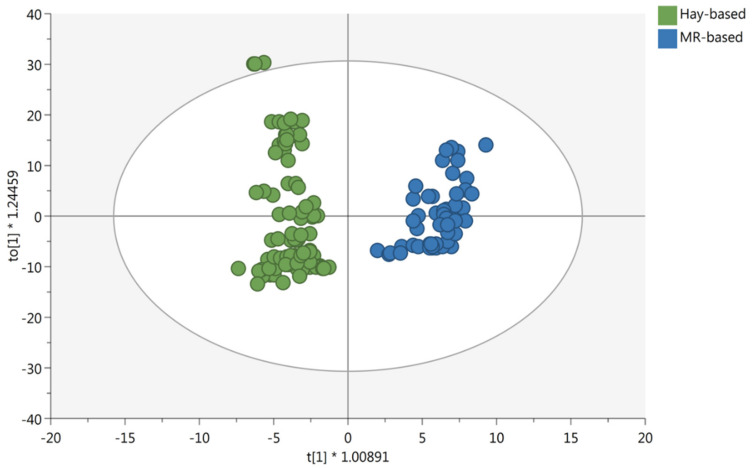
Orthogonal projection to latent structures discriminant analysis (OPLS-DA) considering the milk samples from different feeding regimens (i.e., H-FS: hay-based feeding system; MR-FS: fresh forage/hay-based feeding system).

As can be observed, the metabolomic dataset was able to explain 37.1% of the total variability when considering the two principal components (i.e., PC1 and PC2). Additionally, the score plot revealed that the chemical profile of milk was very similar between the two different sample groups (i.e., hay vs. hay/fresh forage), although a separation trend between the two feeding systems could be highlighted (Figure 1). Therefore, although no distinct information about the specific composition of each diet, untargeted metabolomics demonstrated a fairly good ability to discriminate the chemical fingerprint provided by the fresh forage, thus justifying the further application of a supervised statistical approach to find possible discriminant and marker compounds. Then, the supervised orthogonal projection to latent structures discriminant analysis (OPLS-DA) was produced to inspect and then evaluate the contribution of each milk metabolite for discrimination purposes. OPLS-DA has been successfully used in previous works, based on cheese traceability and when considering the impact of cows’ feeding on the chemical profile of milk intended for processing into cheese [7,12]. Accordingly, by introducing an orthogonal signal correction, this supervised method removes the variation not directly correlated with Y in the X matrix, thus considering only the Y-predictive variation. Furthermore, the method maximizes the covariance existing between the measured data (i.e., MS-peak intensities) and the response variable (i.e., predictive classification based on the feeding system). The OPLS-DA score plot, built considering the feeding system as a class-discriminant parameter, is provided as Figure 2.

As can be observed from the OPLS-DA score plot, the introduction of an orthogonal signal correction allowed for a clear separation between the two different observation groups. Additionally, in our experimental conditions, the OPLS-DA model showed acceptable cross-validation parameters, being R^2^X (cum) = 0.556, R^2^Y (cum) = 0.93 and Q^2^Y = 0.89, whilst the output of permutation test cross-validation (*N* = 200; Supplementary Material) revealed no over-fitting. Furthermore, the misclassification table (Supplementary Material) showed an overall class prediction accuracy of 100%, with a *p*-value 1.10 × 10^−39^. Therefore, the robustness of the OPLS-DA model based on the milk chemical fingerprint was confirmed by noticing no over-fitting and high correlation/prediction abilities.

### 3.2. Marker Compounds of the Feeding System

The variable importance in projection (VIP) of the OPLS-DA model was then used to select the most discriminant metabolites. In particular, the VIP selection method was used to rank the importance of milk metabolites in prediction. This approach allowed us to identify 89 discriminant milk metabolites, possessing a VIP score > 1 and, therefore, driving the hyperspace separation observed (Figure 2). A comprehensive list containing all the metabolites collected in chemical classes is provided in the Supplementary Material. Additionally, for each VIP discriminant compound resulting from the VIP selection method, we evaluated its Log Fold-Change (FC) variation between the two feeding systems, together with its significance (as resulting by ANOVA coupled with Bonferroni False Discovery Rate analysis) and ROC values. The latter were used to describe the robustness of the multivariate statistical analysis. In fact, ROC AUC values represent the measure of how well a VIP marker from the OPLS-DA model can distinguish between the different observation groups.

Overall, Table 1 reports those 20 significant metabolites presenting a VIP score >1, together with their LogFC variation (between Hay/Fresh forage and Hay-based feeding systems), *p*-values, and significant ROC AUC values. It was interesting to notice that, among the 89 VIP discriminant compounds, only 22.5% was found to be a good candidate for discriminating the two feeding regimens. In fact, according to the literature [31], those marker compounds characterized by ROC AUC values > 0.8 are considered fairly good candidates for discrimination purposes between groups. As shown in Table 1, only six compounds were characterized by ROC AUC values higher than 0.8, namely LysoPC(10:0), 3-hexenedioic acid, LysoPE(0:0/22:1(13Z)), benzene-1,2,4-triol, stearoylglycine, and LysoPE(22:1(13Z)/0:0). In this regard, the highest significance as a discriminant marker was recorded for LysoPE(22:1(13Z)/0:0) (being 0.93), and followed by stearoylglycine and benzene-1,2,4-triol (both compounds presenting a value of 0.91).

Looking to the discriminant markers proposed in Table 1, it was possible to notice that 10 compounds were particularly able to explain the differences as related to the addition of forage (i.e., MR-FS) in the cows’ diet. In this regard, the majority of these discriminant metabolites belonged to glycerophospholipids (including lysophosphatidylethanolamines, lysophosphatidylcholines, and phosphatidylcholines), followed by a phenolic metabolite likely related to the forage, such as 5-(3′,4′-Dihydroxyphenyl)-gamma-valerolactone-4′-O-glucuronide and 5alpha-Androstan-3alpha,17beta-diol disulfate (also known as 5a-androstan-3a,17b-diol disulfuric acid). Overall, 5-(3′,4′-Dihydroxyphenyl)-gamma-valerolactone-4′-O-glucuronide belongs to the class of organic compounds known as phenolic glycosides; this compound is reported to be related to a microbial metabolism of flavonoids, as promoted by gut microbiota [32]. In particular, the C-ring fission of flavan-3-ols produces the corresponding diphenylpropan-2-ol and then, this metabolite is further converted into 5-(3′,4′,5′)-tri- and 5-(3′,4′)-Dihydroxyphenyl-gamma-valerolactone in the cases of gallate esters and monomers, respectively. Therefore, gamma-valerolactones and their derivatives (i.e., phenylvaleric acids) have been proposed as exclusive microbial metabolites of flavan-3-ols [33], and likely related to the inclusion of forage in the cow’s diet. Regarding the other marker compounds, in our experimental conditions, it is difficult to find stronger correlations with the feeding system group. For example, 5alpha-Androstan-3alpha, 17beta-diol disulfate is a compound that belongs to the class of organic compounds known as sulfated steroids. This sterol lipid represents an androgen metabolite and possesses a role in the regulation of the hypothalamic–pituitary–adrenal axis (HPA) [34], i.e., a basic reaction of animals to environmental perturbations that threaten homeostasis. Additionally, it has been described as a potent endogenous positive allosteric modulator of the γ–aminobutyric acid (GABA)_A_ receptor, thus potentially being involved in preventing the expression of anxiety and other stress-induced pathologies [35]. Another up-accumulated (LogFC = 1.86) and very significant (VIP score = 2.40 and ROC AUC = 0.91) metabolite characterizing the MR-FS group was N-stearoyl glycine. This compound belongs to the class of organic compounds known as N-acyl-alpha amino acids; these compounds contain an alpha amino acid which bears an acyl group at its terminal nitrogen atom. Thus, stearoylglycine is considered to be a fatty amide lipid molecule. Interestingly, N-acyl-amino acids have emerged in recent years as an important family of endogenous signaling molecules [36]. In fact, they bear many similarities to anandamide, one of the important endocannabinoids, both in chemical structure and in biologic activity, thus possessing both analgesic and anti-inflammatory actions. Finally, the great number of lysophospholipids characterizing the MR-FS group could be related to phospholipolytic phenomena caused by different types of bacterial phospholipases [37]. Overall, it is known that in bovine raw milk, phospholipids are characterized by a great predominance, with a great distribution of phosphatidylcholines (36%), followed by phosphatidylethanolamines (27%), sphingomyelins (22%), phosphatidylinositols (11%), and phosphatidylserines (4%). Additionally, phospholipids and their breakdown products are bioactive compounds involved in cell signaling and have been associated with positive health-promoting effects, such as a reduced risk of neurodegenerative diseases, and antibacterial, anti-inflammatory, and anticancer activities, in addition to a protective role in gastrointestinal mucus [37,38]. Raw milk constitutes an ideal growth medium for different microbes and the main milk constituents also represent potential substrates for a variety of enzymes. In this regard, cold storage represents one of the most used methods to preserve raw milk quality from farms to dairies; however, it does not prevent the growth of psychrotrophic bacteria producing proteases, lipases, and phospholipases that can degrade milk components and cause spoilage [37]. Among this bacterial group, the most important to be cited are *Pseudomonas*, *Acinetobacter*, and *Bacillus*. Additionally, the heat treatments usually carried out in dairies eliminate most of the bacteria load; however, due to their remarkable heat resistance, many of the secreted bacterial enzymes produced by psychrotrophs during cold storage can continue their spoilage actions in both milk and derived dairy products [39]. Therefore, taken together, our findings seem to suggest a great complexity of the markers annotated in providing a clear discrimination between MR- and H-FS groups, likely due to the complexity and the variability of the matrix considered (i.e., milk). Furthermore, considering that in this work, no information is provided regarding the detailed chemical composition of the feeding regimen (i.e., H-FS vs. MR-FS), further investigations appear to be worthwhile in order to assess the importance of these metabolites for discrimination purposes.

### 3.3. Microbial Community Diversity

**Figure 3:**
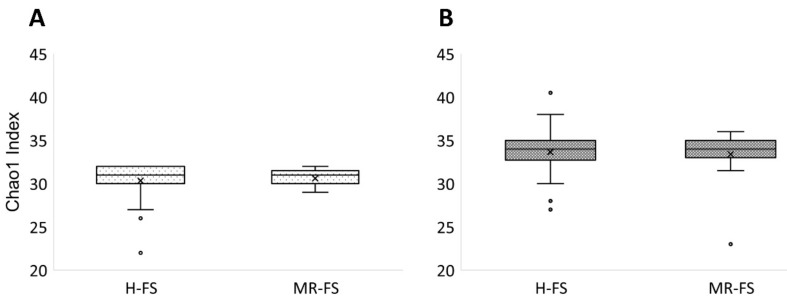
α-diversity plots calculated with the Chao1 index at the genus (**A**) and family (**B**) level.

In this work, we sequenced the 16S rRNA amplicon gene to study the microbial composition and its possible correlations with the metabolomic profile previously reported and resulting from the different feeding regimens. Firstly, we found a substantial variability in the milk samples analyzed, likely due to the potential contamination factors affecting milk in the cowshed. In particular, the α-diversity indices calculated according to the Chao1 index (which considers the observed OTUs, taking into account also those species not observed on the basis of low abundance OTUs) did not reveal a significantly different microbial community in the comparison of the MR-FS vs. the H-FS group at both the taxonomic genus and family level (Figure 3).

**Figure 4:**
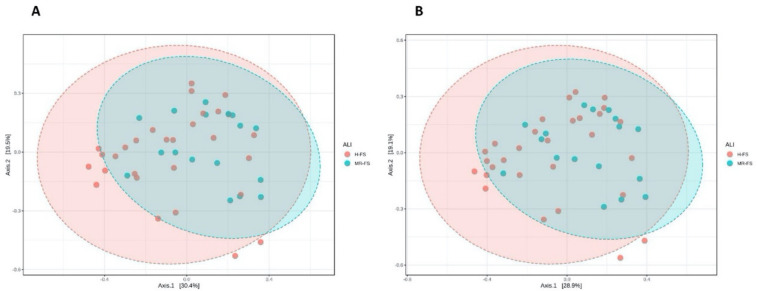
β-diversity plots calculated with PCoA distance matrix and Bray–Curtis index at the family (**A**) and genus (**B**) level.

The same results were recorded when considering the richness and uniformity calculated with the observed Shannon and OTU indices (data not reported), being characterized by no statistical differences between H-FS and MR-FS groups at both taxonomic levels. Additionally, the analysis of the main PCoA components for the β-diversity test did not show strong and significant changes in the OTUs composition between H-FS and MR-FS. Overall, we noted that H-FS was characterized by a greater 95% confidence interval (represented by the red ellipse) when compared with the MR-FS group, thus suggesting a higher variability of those milk samples included in the H-FS group (Figure 4).

Therefore, the classical multivariate statistical analyses showed a low and not significant discrimination potential when considering the two groups based on feeding regimens. Overall, the animals diet directly affects the fecal microbiota [40], whilst the microbial community is influenced by multiple environmental and non-environmental factors [41]. In fact, the most likely scenario is represented by a fecal contamination of the milk, thus resulting in a microbial profile not only directly related to diet. Therefore, the influence of variables related to both management and cleaning systems (not controlled in the present study) becomes strongly significant.

### 3.4. Microbial Community Taxonomy

**Figure 5:**
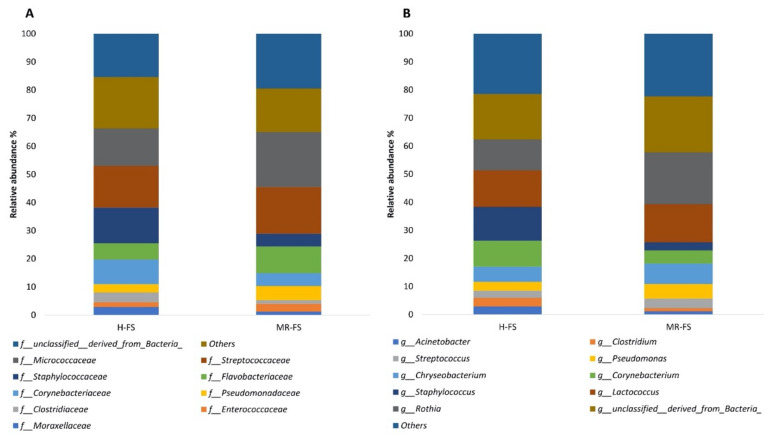
Relative abundance of Top-ten OTUs at the genus (**A**) and family (**B**) level.

Thereafter, taxonomic classification was carried out at both family and gender levels. At the family level (Figure 5), evaluation of the relative abundance of the highest top 10 OTUs revealed that the populations of *Corynebacteriaceae* and *Staphylococcaceae* were, on average, 8% and 4% higher in H-FS samples than in MR-FS samples.

**Figure 6:**
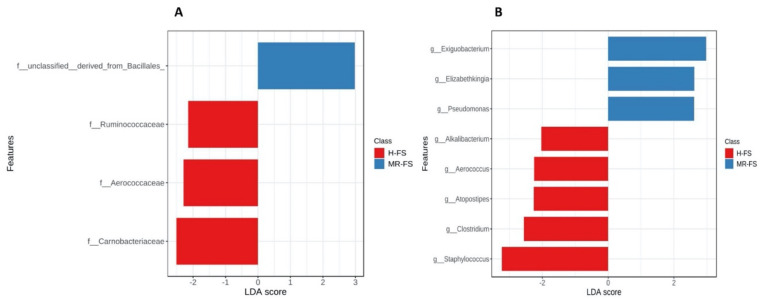
Linear discriminant analysis (LDA) effect size (LEfSe) at the family (**A**) and genus (**B**) taxonomic level. The horizontal bars represent the effect size for each taxon. The length of the bar represents the log10-transformed LDA score, indicated by vertical dotted lines. The red bars represented the significant OTUs of the H-FS group and the blue bars of the MR-FS group.

In contrast, the *Flavobacteriaceae* and *Micrococcaceae* populations were, on average, 3% and 6% smaller in the H-FS samples compared to the MR-FS samples. Additionally, we observed a higher presence of *Micrococcaceae* in the MR-FS samples in the genus *Rothia*, whilst a higher presence of the families *Corynebacteriaceae* and *Staphylococcaceae* in H-FS samples was driven by both *Corynebacterium* and *Staphylococcus* genera, respectively. Interestingly, some previous studies associated the presence of *Corynebacterium* and *Staphylococcus* with a high number of somatic cells and the possibility of occurrence in chewing infections [42,43]. To investigate the significant OTUs for each group, a LEfSe analysis was conducted at both the genus and family level. The LEfSe analysis generated a bar graph (Figure 6) showing those significant OTUs with an LDA score above 2.0 and a p-value below 0.1 (i.e., the default parameters of the MicrobiomeAnalyst). At the family level, *Ruminococcaceae*, *Aerococcaceae*, and *Carnobacteriaceae* were significant for the H-FS group, whilst non-classified OTUs (belonging to the *Bacillales* order) were significant for the MR-FS group. Additionally, *Alkalibacterium*, *Aerococcus*, *Atopostipes*, *Clostridium*, and *Staphylococcus* were found to be significant for the H-FS group, whilst *Exiguobacterium*, *Elizabethkingia,* and *Pseudomonas* were significant only for the MR-FS group.

### 3.5. Correlation between Metabolomic and Metagenomic Profiles

Finally, Pearson’s correlation coefficients were inspected when considering the significant marker compounds (from metabolomics) and the significant microbial families and genera (from metagenomics). Overall, Table 2 reports a summary containing those families/genera establishing the higher number of correlations with the marker compounds (from metabolomics) and vice versa. Furthermore, a comprehensive table reporting each correlation coefficient can be found in the Supplementary Material. At the family level, we found that *Staphylococcaceae*, *Pseudomonadaceae,* and *Dermabacteraceae* established a higher number of significant (*p* < 0.05) correlations with the marker compounds (i.e., nine correlations) when compared with the other microbial families. Interestingly, regarding the significant and validated marker compounds from metabolomics, it was possible to notice that LysoPE (22:1(13Z/0:0)) and LysoPE (0:0/22:1(13Z)) (i.e., marker compounds of the MR-feeding regimen) were able to establish seven and five significant correlations with the microbial families, respectively (Table 2). Furthermore, regarding the correlations at the genus level, we found a predominance of *Pseudomonas* (nine significance correlations), followed by *Staphylococcus* and *Brachynacterium* (both recording eight correlations). Again, when moving to the genus level, we found that LysoPE (22:1(13Z/0:0)) and LysoPE (0:0/22:1(13Z)) were those metabolites establishing the higher number of correlations, being seven and six, respectively.

In this work, great importance for discrimination purposes was highlighted when considering both *Pseudomonas* and *Staphylococcus* genus. This was particularly true when focusing on their taxonomical relevance as provided by LEfSe analysis for the MR-FS and H-FS group, respectively. Overall, it is not clear why *Staphylococcus* and *Pseudomonas* were significant on the basis of the different cattle feeding regimen, but the significant correlations found with some of the metabolomic markers (such as lysophospholipids) seem to suggest some hypotheses. In this regard, *Pseudomonas* being a phospholipolytic bacterial genus [39] finds a better growth environment if the phospholipid content is favorable. Accordingly, as reported in the previous paragraphs, the MR-FS group derived from cows fed with a portion of fresh forage was characterized by a range of up-accumulated phospholipids, such as lysophospholipids. As a possible consequence, we can speculate that a milk rich in phospholipids and *Pseudomonas* may be subjected to certain lipolytic phenomena during the processing of milk into cheese, thus resulting in an undesirable off-flavor for the consumer. On the other hand, when considering *Staphylococcus*, its correlation with those metabolites discriminating H-FS was more complex to describe, considering that *Staphylococcus* is often associated with cows with a history of mastitis, where the milk metabolome is strongly and directly affected by ongoing infections [44]. Therefore, considering that the aim of this work was different from investigation of the health status of dairy cows, we merely observed a significant presence of *Staphylococcus* in response to some up-accumulated metabolites resulting from H-FS regimens.

## 4. Conclusions

In this work, we used a combined untargeted metabolomics/metagenomics approach to discriminate raw milk from dairy cows following a different feeding regimen (i.e., hay-based vs. a mixed ration including hay and forage). The metabolomics-based approach allowed us to confirm distinct chemical signatures when considering both feeding regimens, whilst the taxonomic discrimination was less robust, likely indicating the difficulty in controlling the many factors influencing the milk microbiota (namely stable environment, milking conditions, and fecal contamination). Interestingly, the metabolomic approach allowed us to identify potential markers of the feeding regimens (mainly lysophospholipids and phenolic metabolites deriving from the forage). Regarding metagenomics, it mainly allowed for a descriptive evaluation of the milk microbiota, with LEfSe analysis indicating some relevant taxonomic units, such as *Pseudomonas* for the MR-HF group and *Staphylococcus* for the H-FS group. Additionally, the correlation analysis between the metagenomics and metabolomics-based data revealed that *Pseudomonas* and *Staphylococcus* were those bacterial genera establishing the higher number of correlations with the discriminating metabolites. It was difficult to speculate on the biological keys of these correlations, considering that it was known that the bacterial metabolism in milk sampled at the dairy farm did not have time enough to provide significant differences within the matrix. On the other hand, the potential and positive correlations observed could affect milk in the subsequent processing stages. For example, the positive correlation observed between *Pseudomonas* and phospholipids could be indicative of the phospholipolytic activity promoted by the *Pseudomonas* genus. This observation is worthy of a further research effort, where the putative link could be assessed during the long ripening time of this cheese. Therefore, taken together, our findings demonstrated the suitability and the potential of untargeted metabolomics to unveil the chemical markers of milk as related to the cow’s feeding regimen. However, the highest possible number of fixed variables able to affect the milk microbiota from the stable to the traditional upturned bell-shaped copper vats should be deeply and better considered.

## Figures and Tables

**Table 1:** Most discriminant marker compounds characterizing the comparison of MR-FS vs. H-FS. Each compound is reported with its VIP score (from the OPLS-DA supervised modelling), LogFC value, *p*-value, and ROC AUC value. FDR = false discovery rate.

Metabolite	VIP Score (OPLS-DA)	LogFC Value (MR-FS vs. H-FS)	*p*-Value (FDR)	ROC AUC
PA(16:0/18:1(11Z))	1.13	1.11	1.54 × 10^−2^	0.65
DG(22:0/0:0/18:2n6)	1.01	−0.72	5.70 × 10^−3^	0.66
5alpha-Androstan-3alpha,17beta-diol disulfate	1.32	0.66	3.10 × 10^−3^	0.67
DG(24:1(15Z)/15:0/0:0)	1.10	−0.94	5.00 × 10^−3^	0.68
PC(16:0/14:1(9Z))	1.43	−1.22	3.12 × 10^−3^	0.71
PC(P-18:0/14:1(9Z))	1.47	2.31	5.04 × 10^−4^	0.72
PC(P-14:0/18:1(9Z))	1.41	1.85	1.61 × 10^−3^	0.73
SM(d18:1/12:0)	1.51	−1.45	8.19 × 10^−6^	0.76
DG(22:2n6/0:0/22:4n6)	1.02	−1.91	1.63 × 10^−7^	0.78
5-(3′,4′-Dihydroxyphenyl)-gamma-valerolactone-4′-*O*-glucuronide	1.41	1.20	1.49 × 10^−3^	0.78
DG(22:0/18:4(6Z,9Z,12Z,15Z)/0:0)	1.75	−2.54	2.01 × 10^−6^	0.79
SM(d16:0/17:1(10Z))	1.70	−2.53	6.44 × 10^−8^	0.79
DG(20:2n6/0:0/20:2n6)	1.63	−1.32	6.17 × 10^−4^	0.79
PC(P-18:1(9Z)/14:1(9Z))	1.60	1.65	2.53 × 10^−9^	0.79
LysoPC(10:0)	1.93	0.86	9.19 × 10^−7^	0.85
3-Hexenedioic acid	2.26	−1.44	6.01 × 10^−13^	0.89
LysoPE(0:0/22:1(13Z))	2.45	2.62	1.02 × 10^−6^	0.89
Benzene-1,2,4-triol	2.43	−1.49	1.32 × 10^−16^	0.91
N-stearoyl glycine	2.40	1.86	1.28 × 10^−21^	0.91
LysoPE(22:1(13Z)/0:0)	2.53	2.61	5.83 × 10^−9^	0.93

Abbreviations: PA (phosphatidic acid); DG (diacylglycerol); PC (phosphatidylcholine); SM (Sphingomyelin); LysoPC (Lysophosphatidylcholine); LysoPE (lysophosphatidylethanolamine).

**Table 2:** Number of significant correlations established by bacterial families and genera with the significant variables importance in projection (VIP) metabolites from untargeted metabolomic profiling.

Family	Number of Significant Correlations (*p* < 0.05)
*Staphylococcaceae*	9
*Pseudomonadaceae*	9
*Dermabacteraceae*	9
*Streptococcaceae*	7
***Genus***	
*Pseudomonas*	9
*Staphylococcus*	8
*Brachybacterium*	8
*Macrococcus*	7
**VIP metabolites**	
LysoPE(22:1(13Z)/0:0)	7 (at both family and genus level)
LysoPE(0:0/22:1(13Z))	6 (at genus level); 5 (at family level)

## Data Availability

Not applicable.

## Supplementary Materials

The following are available online at https://www.mdpi.com/2304-8158/10/1/109/s1, Table S1: Metabolomic and metagenomic dataset, together with validation parameters of OPLS-DA modelling and Pearson’s correlation coefficients when considering metabolomics and metagenomics significant data.
